# Reference library for microbial source tracking in the mid-Atlantic United States

**DOI:** 10.1128/MRA.00674-23

**Published:** 2023-11-30

**Authors:** Malique Bowen, Ibrahim F. farag, Christopher R. Main, Jennifer F. Biddle

**Affiliations:** 1 School of Marine Science and Policy, University of Delaware, Lewes, Delaware, USA; 2 Delaware Department of Natural Resources and Environmental Control, Dover, Delaware, USA; Queens College, Queens, New York, USA

**Keywords:** microbial source tracking, reference library

## Abstract

Microbial source tracking can determine fecal contamination but requires a relevant, sizable reference library for analysis. We provide a reference library of 100+ fecal microbiome samples relevant to mid-Atlantic United States ecosystems. Included are wild and domesticated fauna, wastewater, and septic samples applicable to Delaware source tracking studies.

## ANNOUNCEMENT

Accepted methods for monitoring water quality in surface waters focus on quantifying cultivable fecal indicator bacteria (FIB; i.e., *Escherichia coli* and *Bacteroides* spp.) (1). However, the quantification of traditional FIB is limited by the inability to distinguish the origin of fecal pollution (2). Microbial source tracking (MST) uses a cultivation-independent, sequence-based reference library to compare the likelihood of a source’s contribution to a sink; thus, MST can serve as an effective tool in identifying potential origins of fecal contamination in watersheds (2). Delaware is home to major watersheds that drain water from areas of mixed land use. Building a reference library relevant to Delaware ecosystems for use in MST studies could aid in maintaining and protecting water quality in the mid-Atlantic region of the United States.

Fecal material was collected throughout southern Delaware from nine species of domestic fauna (i.e., farm animals and pets), influent septic waste, and a wastewater treatment outflow and was stored at −80°C until processing (Table 1). Wildlife biologists visually identified and collected wild scat, representing a total of seven species, and samples were also stored at −80°C until processing. Fecal matter (1 g) was homogenized with 100 mL of 1× sterile phosphate-buffered saline to create a slurry to ensure microbial homogeneity across the individual samples followed by DNA extraction using the Qiagen DNeasy PowerSoil Pro kit (Germantown, Maryland). Extracted DNA was sent to the UCONN MARS facility for amplicon sequencing of the 16S rRNA gene in the V4 region (primers 515F and 806R) using the Illumina MiSeq paired-end sequencing platform via the version 2 2 × 250 base pair kit (Illumina) following their standard protocol. Forward and reverse sequences were quality checked, and only the forward sequence reads passed quality check and were further processed using the 16S rRNA gene analysis pipeline implemented in MOTHUR version 1.46.1 (3). Sequences were trimmed to a 130- to 200-bp range; ambiguous nucleotides were removed; and operational taxonomic units (OTUs) with a 3% dissimilarity were created. OTUs were aligned and classified using the Silva SSU database, version 138 (4).

**TABLE 1:** Metadata, sequence read counts, and OTUs of fecal samples used to build reference library

Filename	Type	Diet	Environment	Collection period	Raw sequence reads	Sequence reads after quality check	OTUs
2002019A.gz	n/a^*a*^	n/a	Human	Winter 2020	34,681	34,681	7,369
2002019B.gz	n/a	n/a	Human	Winter 2020	37,217	37,217	8,193
2002019C.gz	n/a	n/a	Human	Winter 2020	43,871	43,871	10,334
2002034A.gz	n/a	n/a	Human	Winter 2020	104,238	104,238	18,425
2002034B.gz	n/a	n/a	Human	Winter 2020	67,934	67,934	12,328
2002034C.gz	n/a	n/a	Human	Winter 2020	58,747	58,747	10,179
Bull1.gz	Domestic	Herbivore	Domestic	Summer 2018	40,700	40,700	6,988
Bull2.gz	Domestic	Herbivore	Domestic	Summer 2018	27,595	27,595	6,272
Bull3.gz	Domestic	Herbivore	Domestic	Summer 2018	46,416	46,416	12,231
Bull4.gz	Domestic	Herbivore	Domestic	Summer 2018	50,537	50,537	12,127
Bull5.gz	Domestic	Herbivore	Domestic	Summer 2018	49,027	49,027	10,022
Bull6.gz	Domestic	Herbivore	Domestic	Summer 2018	57,126	57,126	11,469
Bull7.gz	Domestic	Herbivore	Domestic	Summer 2018	54,458	54,458	9,237
Bull8.gz	Domestic	Herbivore	Domestic	Summer 2018	43,761	43,761	9,679
Bull10.gz	Domestic	Herbivore	Domestic	Summer 2018	35,616	35,616	7,796
Cat1.gz	Domestic	Carnivore	Domestic	Fall 2020	32,396	32,313	1,933
Cat2.gz	Domestic	Carnivore	Domestic	Fall 2020	40,771	40,617	2,577
Cat4.gz	Domestic	Carnivore	Domestic	Fall 2020	29,320	29,057	1,283
Cat5.gz	Domestic	Carnivore	Domestic	Fall 2020	31,804	31,471	1,289
Cat6.gz	Domestic	Carnivore	Domestic	Fall 2020	21,153	21,153	1,133
Cat7.gz	Domestic	Carnivore	Domestic	Fall 2020	14,538	14,538	765
Chicken1.gz	Domestic	Omnivore	Domestic	Summer 2018	50,464	50,464	6,006
Chicken2.gz	Domestic	Omnivore	Domestic	Summer 2018	60,187	60,187	5,100
Chicken3.gz	Domestic	Omnivore	Domestic	Summer 2018	57,538	57,538	10,604
Chicken4.gz	Domestic	Omnivore	Domestic	Summer 2018	63,863	63,863	5,007
Chicken5.gz	Domestic	Omnivore	Domestic	Summer 2018	35,208	35,208	7,646
Chicken6.gz	Domestic	Omnivore	Domestic	Summer 2018	44,563	44,563	10,857
Chicken7.gz	Domestic	Omnivore	Domestic	Summer 2018	53,049	53,049	7,869
Chicken8.gz	Domestic	Omnivore	Domestic	Summer 2018	57,379	57,379	10,162
Chicken9.gz	Domestic	Omnivore	Domestic	Summer 2018	62,498	62,498	13,946
Chicken10.gz	Domestic	Omnivore	Domestic	Summer 2018	51,747	51,747	5,555
Deer1.gz	Wild	Herbivore	Wild	Fall 2020	27,060	27,060	5,051
Deer2.gz	Wild	Herbivore	Wild	Fall 2020	90,728	90,728	12,258
Deer3.gz	Wild	Herbivore	Wild	Fall 2020	76,542	76,542	8,960
Deer4.gz	Wild	Herbivore	Wild	Fall 2020	58,796	58,796	7,579
Deer5.gz	Wild	Herbivore	Wild	Fall 2020	72,712	72,712	6,721
Deer6.gz	Wild	Herbivore	Wild	Fall 2020	74,753	74,753	9,058
Deer7.gz	Wild	Herbivore	Wild	Fall 2020	86,743	86,743	10,285
Deer8.gz	Wild	Herbivore	Wild	Spring 2021	58,735	58,735	8,444
Deer9.gz	Wild	Herbivore	Wild	Spring 2021	64,348	64,348	7,483
Deer10.gz	Wild	Herbivore	Wild	Spring 2021	79,874	79,874	7,457
Deer11.gz	Wild	Herbivore	Wild	Spring 2021	70,732	70,732	9,056
Dog1.gz	Domestic	Omnivore	Domestic	Fall 2020	281,618	28,149	1,440
Dog2.gz	Domestic	Omnivore	Domestic	Fall 2020	27,266	27,254	856
Dog3.gz	Domestic	Omnivore	Domestic	Fall 2020	31,654	31,629	1,634
Dog4.gz	Domestic	Omnivore	Domestic	Fall 2020	44,933	44,858	1,312
Dog5.gz	Domestic	Omnivore	Domestic	Fall 2020	18,812	18,731	1,418
Dog6.gz	Domestic	Omnivore	Domestic	Fall 2020	28,460	28,409	3,039
Dog7.gz	Domestic	Omnivore	Domestic	Fall 2020	25,869	25,843	1,405
Duck1.gz	Wild	Omnivore	Wild	Spring 2021	43617	43,617	8,548
Duck2.gz	Wild	Omnivore	Wild	Spring 2021	50,308	50,308	14,104
Duck3.gz	Wild	Omnivore	Wild	Spring 2021	60,346	60,346	6,964
Duck4.gz	Wild	Omnivore	Wild	Spring 2021	50,423	50,423	11,099
Duck5.gz	Wild	Omnivore	Wild	Spring 2021	70,325	70,325	15,426
Fox1.gz	Wild	Omnivore	Wild	Fall 2019	44,166	44,166	8,377
Fox2.gz	Wild	Omnivore	Wild	Fall 2019	61,362	61,362	11,846
Fox3.gz	Wild	Omnivore	Wild	Fall 2019	98,459	98,459	5,776
Fox4.gz	Wild	Omnivore	Wild	Fall 2020	81,750	81,750	4,973
Fox5.gz	Wild	Omnivore	Wild	Fall 2020	128,795	128,795	6,856
Fox6.gz	Wild	Omnivore	Wild	Fall 2020	21,065	21,022	1,655
Goat1.gz	Domestic	Herbivore	Domestic	Summer 2018	34,906	34,906	4,425
Goat2.gz	Domestic	Herbivore	Domestic	Summer 2018	39,326	39,326	6,309
Goat3.gz	Domestic	Herbivore	Domestic	Summer 2018	32,177	32,177	6,419
Goat4.gz	Domestic	Herbivore	Domestic	Summer 2018	53,833	53,833	11,013
Goat5.gz	Domestic	Herbivore	Domestic	Summer 2018	51,388	51,388	12,789
Goat6.gz	Domestic	Herbivore	Domestic	Summer 2018	56,596	56,596	12,897
Goat7.gz	Domestic	Herbivore	Domestic	Summer 2018	47,773	47,773	11,344
Goat9.gz	Domestic	Herbivore	Domestic	Summer 2018	56,482	56,482	12,010
Goat10.gz	Domestic	Herbivore	Domestic	Summer 2018	52,401	52,401	10,245
Goose1.gz	Wild	Herbivore	Wild	Fall 2019	34,929	34,906	4,225
Goose2.gz	Wild	Herbivore	Wild	Fall 2019	40,060	39,326	6,309
Goose3.gz	Wild	Herbivore	Wild	Fall 2019	33,144	32,177	6,419
Goose4.gz	Wild	Herbivore	Wild	Fall 2019	55,711	53,833	11,013
Goose5.gz	Wild	Herbivore	Wild	Fall 2019	39,837	39,734	5,421
Goose6.gz	Wild	Herbivore	Wild	Fall 2020	40,803	40,685	5,322
Goose7.gz	Wild	Herbivore	Wild	Fall 2020	20,117	20,063	3,492
Goose8.gz	Wild	Herbivore	Wild	Fall 2020	28,156	28,098	5,034
Goose9.gz	Wild	Herbivore	Wild	Fall 2020	24,667	24,631	4,196
Goose10.gz	Wild	Herbivore	Wild	Fall 2020	15,346	15,318	1,681
Horse1.gz	Domestic	Herbivore	Domestic	Summer 2018	40,790	40,790	11,077
Horse2.gz	Domestic	Herbivore	Domestic	Summer 2018	44,590	44,590	8,909
Horse5.gz	Domestic	Herbivore	Domestic	Summer 2018	44,251	44,251	10,028
KCWTP.gz	n/a	n/a	Human	Winter 2021	67,994	67,994	7,365
KCWWTP.gz	n/a	n/a	Human	Winter 2021	36,175	36,175	4,876
Pig1.gz	Domestic	Omnivore	Domestic	Summer 2018	57,221	57,221	13,763
Pig2.gz	Domestic	Omnivore	Domestic	Summer 2018	64,693	64,693	12,101
Pig3.gz	Domestic	Omnivore	Domestic	Summer 2018	51,590	51,590	11,491
Pig4.gz	Domestic	Omnivore	Domestic	Summer 2018	43,115	43,115	5,056
Pig5.gz	Domestic	Omnivore	Domestic	Summer 2018	61,131	61,131	11,766
Pig6.gz	Domestic	Omnivore	Domestic	Summer 2018	4,689	4,689	1,542
Pig7.gz	Domestic	Omnivore	Domestic	Summer 2018	57,576	57,576	8,400
Pig8.gz	Domestic	Omnivore	Domestic	Summer 2018	47,643	47,643	5,893
Pig9.gz	Domestic	Omnivore	Domestic	Summer 2018	46,526	46,526	10,099
Pig10.gz	Domestic	Omnivore	Domestic	Summer 2018	39,185	39,155	3,895
Rabbit1.gz	Domestic	Herbivore	Domestic	Summer 2018	62,066	62,066	6,866
Rabbit2.gz	Domestic	Herbivore	Domestic	Summer 2018	47,210	47,210	7,832
Rabbit3.gz	Domestic	Herbivore	Domestic	Summer 2018	55,650	55,650	12,259
Rabbit4.gz	Domestic	Herbivore	Domestic	Summer 2018	54,280	54,280	10,138
Rabbit5.gz	Domestic	Herbivore	Domestic	Summer 2018	44,755	44,755	9,432
Raccoon1.gz	Wild	Omnivore	Wild	Fall 2019	103	103	94
Raccoon2.gz	Wild	Omnivore	Wild	Fall 2019	28,784	28,733	1,604
Raccoon3.gz	Wild	Omnivore	Wild	Fall 2019	31,700	31661	1,412
Raccoon5.gz	Wild	Omnivore	Wild	Fall 2019	21,336	21325	560
Raccoon6.gz	Wild	Omnivore	Wild	Fall 2019	8,375	8,371	753
Raccoon7.gz	Wild	Omnivore	Wild	Fall 2019	36,929	36,909	1,376
Raccoon8.gz	Wild	Omnivore	Wild	Summer 2021	10,452	10,284	346
Raccoon9.gz	Wild	Omnivore	Wild	Summer 2021	27,010	26,958	1,953
Raccoon10.gz	Wild	Omnivore	Wild	Summer 2021	114	101	31
Raccoon11.gz	Wild	Omnivore	Wild	Summer 2021	31,080	31,066	1,238
Sheep1.gz	Domestic	Herbivore	Domestic	Summer 2018	32,824,	32,824	3,570
Sheep2.gz	Domestic	Herbivore	Domestic	Summer 2018	68,228	68,228	18,285
Sheep3.gz	Domestic	Herbivore	Domestic	Summer 2018	59,801	59,801	8,912
Sheep4.gz	Domestic	Herbivore	Domestic	Summer 2018	39,548	39,548	7,765
Sheep5.gz	Domestic	Herbivore	Domestic	Summer 2018	47,190	47,190	9,195
Sheep6.gz	Domestic	Herbivore	Domestic	Summer 2018	39,806	39,806	7,511
Sheep7.gz	Domestic	Herbivore	Domestic	Summer 2018	51,540	51,540	11,673
Sheep8.gz	Domestic	Herbivore	Domestic	Summer 2018	42,534	42,534	7,570
Sheep9.gz	Domestic	Herbivore	Domestic	Summer 2018	34,720	34,720	8,579
Sheep10.gz	Domestic	Herbivore	Domestic	Summer 2018	57,674	57,674	11,960
Squirrel1.gz	Wild	Omnivore	Wild	Fall 2019	44,839	44,839	8,863
Squirrel2.gz	Wild	Omnivore	Wild	Fall 2019	26,631	26,590	2,090
Squirrel3.gz	Wild	Omnivore	Wild	Fall 2019	34,273	34,258	2,274
Turkey1.gz	Wild	Omnivore	Wild	Fall 2019	40,857	40,857	9,310
Turkey2.gz	Wild	Omnivore	Wild	Fall 2019	33,398	33,398	6,651

^*a*^ n/a, not applicable.

We used 124 fecal sources to build a regional reference library for MST studies. Of the 18 fecal hosts, 9 were domestic, including pets and agricultural livestock that were freshly sampled; 7 were wild from natural settings, including aged material; and 2 were human relevant samples from septic systems and influent wastewaters (Table 1). This data set spans domestic and wild habitats, diet types (herbivore and omnivore), and species. This reference library should be applicable for MST studies of waterways, lakes, and ponds in the mid-Atlantic region of the United States.

## Data Availability

Data available under BioProject number PRJNA996235.
